# Two episodes of Taravana syndrome in a breath‐hold diver with hyperhomocysteinemia

**DOI:** 10.1002/ccr3.1479

**Published:** 2018-03-08

**Authors:** Giuseppe Accurso, Andrea Cortegiani, Sabrina Caruso, Oriana Danile, Domenico Garbo, Pasquale iozzo, Filippo Vitale, Santi Maurizio Raineri, Cesare Gregoretti, Antonino Giarratano

**Affiliations:** ^1^ Department of Biopathology and Medical Biotechnologies (DIBIMED) Section of Anesthesia, Analgesia, Intensive Care and Emergency Policlinico Paolo Giaccone University of Palermo Palermo Italy

**Keywords:** Breath‐hold diving, dysbaric accidents, hyperhomocysteinemia, Taravana syndrome

## Abstract

Taravana syndrome is a rare dysbaric disease characterized by neurologic signs and symptoms. Differently from others decompression illness, it has unspecified pathophysiology and unclear predisposing factors. Our cases suggest that thrombophilic state due to hyperhomocysteinemia could increase the risk to develop Taravana syndrome.

## Introduction

Breath‐hold (BH) diving can cause underwater accidents including the rare Taravana syndrome (TS). Its predisposing factors are unclear. Hyperhomocysteinemia is a risk factor for stroke. Its role in dysbaric accidents has never been evaluated. We report two episodes of TS occurred in a young spearfishing champion affected by hyperhomocysteinemia.

Repetitive breath‐hold (BH) diving can cause underwater accidents including the rare Taravana syndrome (TS). Firstly reported by Cross in the 1965, it is characterized by neurologic disorders such as dizziness, cross‐sensory numbness, nausea, euphoria, dysarthria, hemiparesis, unconsciousness, and even sudden death 1. In some cases, symptoms are sudden, while in other cases they appear 1–2 h later, depending on the dive profile 2. Although some cases have been reported in the literature, the pathophysiology and the predisposing factors of this syndrome are still unclear 2. Hyperhomocysteinemia has been found to be a risk factor for cardiovascular diseases 3. Nonetheless its role in TS has never been evaluated.

We report two episodes of TS occurred in the same young male spearfishing champion affected by hyperhomocysteinemia treated with recompression treatment at the Hyperbaric Oxygen Therapy (HBOT) Department of the University Hospital Policlinico Paolo Giaccone, Palermo, Italy, in November 2010 and July 2016.

## Cases Description

A 38‐year‐old man was admitted to the emergency department the day after a dive session on July 2010. On physical examination, he showed left buccal rhyme deviation and homolatreal facial paresthesia that had appeared at the end of the diving session. His past medical history included no smoking, dust allergy, celiac disease without any ongoing medication.

He started diving at the age of 14, and he had been practicing competitive spearfishing for 10 years.

Data retrieved from his personal logger shoved a total diving time of 7 h with an average water temperature of 17°C. He ran 100–120 dives at a maximum depth of 40 m with an apnea time not exceeding 1.4 min.

**Figure 1:**
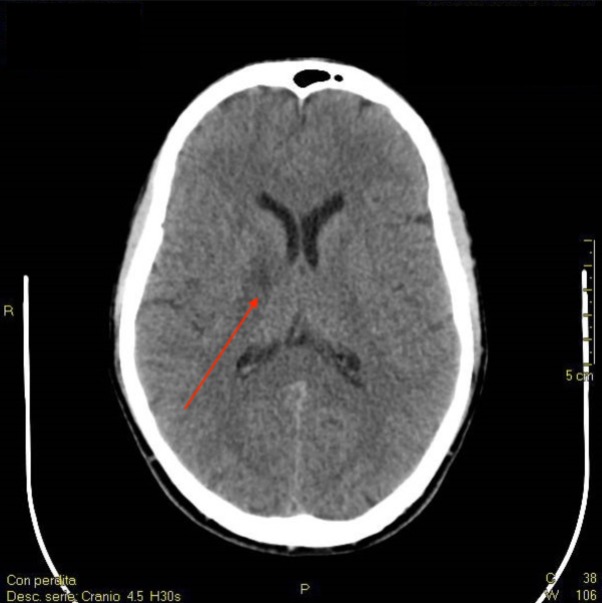
Brain CT scan showing a slight hypodense area in the right internal capsula (red arrow).

His vital parameters were normal (noninvasive blood pressure 143/88 mmHg and heart rate 80/min). The neurological assessment revealed phasic well‐oriented patient, a Glasgow Coma Scale value of 15/15, bilaterally isochoric, isocyclic, pupils, and absence of other neurological deficits. He was eupnic without any pathological sounds on chest examination. Blood works, including first‐line coagulation tests, and twelve‐lead ECG were normal. A brain computerized tomography (CT) scan without contrast showed a slight hypodense area in the right internal capsule (Fig. 1).

On suspicion of TS, the patient was referred to the HBOT Unit to start a prompt treatment. Recompression treatment consisted of eight sessions using US NAVY table 6 with full regression of signs 4. Echocolor Doppler of supra‐aortic vessels was then performed; it did not reveal any abnormality. A transthoracic echocardiography (with contrast agent injection) looking for patency of foramen ovale was also performed; no signs of patency were detected. He restarted agonistic activity after 5 months.

The second episode happened in July 2016. Data retrieved from his personal logger showed a total diving time of 4 h with an average water temperature between 22 and 23°C. He ran 60 dives, at a maximum depth of 40 m, with an apnea time that was not exceeding 1.4 min.

At the end of the dive, he started to complain of tingling on the left arm, left leg paresthesia, and asthenia. He decided to dive again, descending to about 5 m, breathing therapeutic oxygen for 5–8 min. Back to surface, he recovered the functionality and sensitivity of left arm and leg, but he continued to complain of profound asthenia.

**Figure 2:**
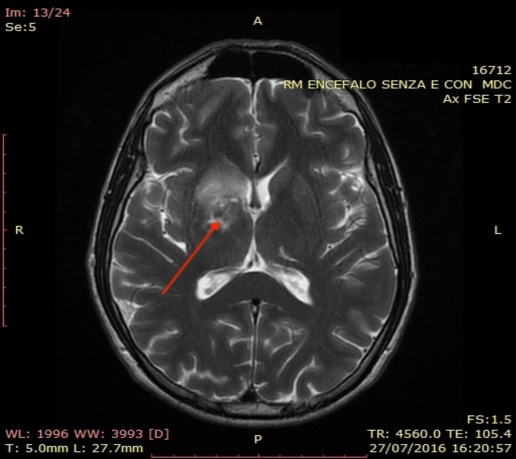
Brain MRI (T2‐weighted sequence). The arrows point to n hyperintensity area mainly involving thalamic and lenticular caudate with extension to internal and periventricular capsule. It is likely to be a subacute ischemic lesion. Day 2 after second episode of diving session.

After about 24 h, he showed left deviation of the buccal rhyme. He was then referred to the nearest ED. A brain CT scan without contrast showed hypodensity on the nucleus of the right thalamus associated with edema. A brain magnetic resonance (MRI) confirmed a lesion in thalamic and lenticular caudate with extension to internal and periventricular capsule compatible with subacute ischemic lesion (Fig. 2). During hospitalization, the patient underwent echocolor Doppler of supra‐aortic vessels which was normal. Laboratory tests for thrombophilic screening were then carried out. High level of homocysteine (28.59 *μ*mol/L) was found. He promptly underwent a session of recompression treatment (US NAVY table 6), with prompt regression of signs and symptoms. Two further subsequent sessions were performed during the following days 4. Acetylsalicylate acid 100 mg/os daily was then prescribed as a prophylactic treatment.

After been discharged 10 days later, he restarted his diving activity after 4 months. No other accidents occurred.

## Discussion

Our findings may suggest that BH diving, in associations with a thrombophilic state due to hyperhomocysteinemia, may be associated with TS. In our case, the diagnosis of TS was suggested by the type of dives and congruent neurological findings, namely sensory numbness or motor weakness on one side, transient neurological deficits that resolve, even without treatments, vertigo, fatigue, nausea, and behavioral disorders 2, 5, 6.

The development of TS depends on several factors, such as the speed of descent, depth, the pre‐apneic hyperventilation, and postapneic asphyxia, the use of weights, assisted‐ascent, and total diving time. The pathophysiology of this syndrome is still controversial, as damage by nitrogen microbubbles is the most credible mechanism. Although the most important factors seem to be the surface intervals and the numbers of dives, all factors influencing the nitrogen distribution and endothelial metabolism could damage physiological functions of the brain. While a single dive does not put the divers at risk of developing TS, the risk increases with more repetitive dives, especially if repeated over time and with short surface intervals due to the increased blood nitrogen tension. When a BH diver performs repetitive and prolonged deep dives, nitrogen silent microbubbles may accumulate in tissues, passing from the peripheral tissues to the pulmonary circulation 2, 7. In cerebral circulation, they impair the blood–brain barrier. Potential causes of developing TS may be right‐to‐left shunt or barotrauma due to with alveolar–capillary membrane disruption. Moreover, aggregation of microbubbles may cause cerebral embolism. The typical neurological lesions of Taravana usually occur in the brain while sparing the spinal cord, which is involved more frequently in compressed‐air decompression illness 7, 8. The nitrogen microbubbles may be responsible for brain damage with both mechanical and biochemical pathological mechanisms: They may reduce the blood flow by a mechanic occlusion of blood vessels and create a microvascular inflammatory reaction of variable entity 9. The main pathophysiological consequences 2 are brain hypoxia, ischemia, and parenchymal damage 7.

After the first episode, the anatomic site of the CT scan brain lesion was likely to be related to chronic blood flow deficit (Fig. 1), which is uncommon in a young athlete. On the other hand, brain MRI showed a typical lesion of TS (Fig. 2), which involves terminal blood flow areas 2, 7. Our patient was a healthy, young, and experienced BH diver. The dive sessions before both episodes presented normal characteristics without predisposing events. The patient did not present atheromatous plaques or abnormalities of supra‐aortic vessels. Moreover, transthoracic echocardiography with contrast agent did not show any sign of patency of foramen ovale. The only altered finding was hyperhomocysteinemia. However, one limitation of our case is that the patient did not perform tests for platelets function. Many studies identified a strong, independent, and dose‐related association between elevated homocysteine and cardiovascular diseases, including stroke 3, 10. Our case may suggest that BH diving, in certain conditions, in associations with a thrombophilic state due to hyperhomocysteinemia, may cause neurologic ischemia and TS with different pathophysiological mechanisms usually involved in dysbaric accidents 1, 2, 8. Moreover, as thrombophilic screening is not usual for divers, this association may be underrated.

## Conclusion

The pathophysiology of TS in BH divers is still unclear. Our case may suggest an association between thrombophilic state due to hyperhomocysteinemia and the development of neurologic damage in BH divers. As thrombophilic screening is not usual for divers, this association may be underestimated. Further research is needed to evaluate this association and to eventually develop a screening/diagnostic algorithm in BH divers.

## Authorship

GA, AC, DG, SMR, CG, AG: treated the patients, helped retrieving the data, conceived the content of the manuscript. SC, OD, PI, FV: helped retrieving the data, helped writing the manuscript. GA, AC, CG: wrote the manuscript. All authors read and approved the final version of the manuscript.

## Conflict of Interest

None declared.
